# *catena*-Poly[[dichloridomercury(II)]-μ-{*N*-[(*E*)-pyridin-2-yl­methyl­idene-κ*N*]pyridin-3-amine-κ^2^ *N* ^1^:*N* ^3^}]

**DOI:** 10.1107/S1600536812035775

**Published:** 2012-09-05

**Authors:** Ali Mahmoudi, Saeed Dehghanpour, Leila Najafi, Mohammad Khalafbeigi

**Affiliations:** a Department of Chemistry, Islamic Azad University University, Karaj, Iran; b Department of Chemistry, Alzahra University, Tehran, Iran

## Abstract

In the title coordination polymer, [HgCl_2_(C_11_H_9_N_3_)]_*n*_, the Hg^II^ ion is coordinated by three N atoms from two *N*-[(*E*)-pyridin-2-yl­methyl­idene]pyridin-3-amine (*L*) ligands and two chloride anions in a distorted trigonal–bipyramidal geometry. The two pyridine rings in *L* form a dihedral angle of 50.0 (2)°. *L* ligands bridge adjacent HgCl_2_ units into polymeric chains propagating in [010]. The crystal packing is further stabilized by weak inter­molecular C—H⋯Cl hydrogen bonds and π–π inter­actions between the pyridine rings, with a centroid–centroid separation of 3.529 (9) Å.

## Related literature

For related structures and applications of coordination polymers, see: Moulton & Zaworotko (2001 ▶); Fei *et al.* (2000 ▶). For the synthesis of the ligand and the index of trigonality, see: Dehghanpour *et al.* (2012 ▶).
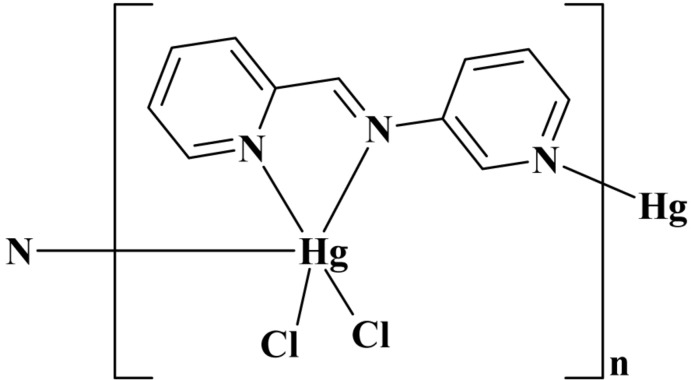


## Experimental

### 

#### Crystal data

[HgCl_2_(C_11_H_9_N_3_)]
*M* *_r_* = 454.70
Monoclinic, 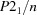

*a* = 7.5645 (5) Å
*b* = 13.1057 (9) Å
*c* = 12.7017 (5) Å
β = 96.077 (4)°
*V* = 1252.15 (13) Å^3^
*Z* = 4
Mo *K*α radiation
μ = 12.70 mm^−1^
*T* = 150 K
0.15 × 0.08 × 0.02 mm

#### Data collection

Nonius KappaCCD diffractometer
Absorption correction: multi-scan (*SORTAV*; Blessing, 1995 ▶) *T*_min_ = 0.566, *T*_max_ = 0.889
8560 measured reflections
2837 independent reflections
2164 reflections with *I* > 2σ(*I*)
*R*_int_ = 0.059

#### Refinement

*R*[*F* ^2^ > 2σ(*F* ^2^)] = 0.042
*wR*(*F* ^2^) = 0.116
*S* = 1.09
2837 reflections
154 parameters
H-atom parameters constrained
Δρ_max_ = 2.45 e Å^−3^
Δρ_min_ = −3.10 e Å^−3^

### 

Data collection: *COLLECT* (Nonius, 2002 ▶); cell refinement: *DENZO-SMN* (Otwinowski & Minor, 1997 ▶); data reduction: *DENZO-SMN*; program(s) used to solve structure: *SIR92* (Altomare *et al.*, 1994 ▶); program(s) used to refine structure: *SHELXTL* (Sheldrick, 2008 ▶); molecular graphics: *PLATON* (Spek, 2009 ▶); software used to prepare material for publication: *SHELXTL*.

## Supplementary Material

Crystal structure: contains datablock(s) I, global. DOI: 10.1107/S1600536812035775/cv5327sup1.cif

Structure factors: contains datablock(s) I. DOI: 10.1107/S1600536812035775/cv5327Isup2.hkl

Additional supplementary materials:  crystallographic information; 3D view; checkCIF report

## Figures and Tables

**Table 1:** Hydrogen-bond geometry (Å, °)

*D*—H⋯*A*	*D*—H	H⋯*A*	*D*⋯*A*	*D*—H⋯*A*
C4—H4*A*⋯Cl2^i^	0.95	2.82	3.700 (8)	154
C6—H6*A*⋯Cl2^i^	0.95	2.79	3.666 (7)	154
C10—H10*A*⋯Cl2^ii^	0.95	2.83	3.545 (8)	132

Symmetry codes: (i) 
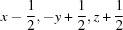
; (ii) 
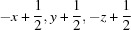
.

**Fig. 1.**
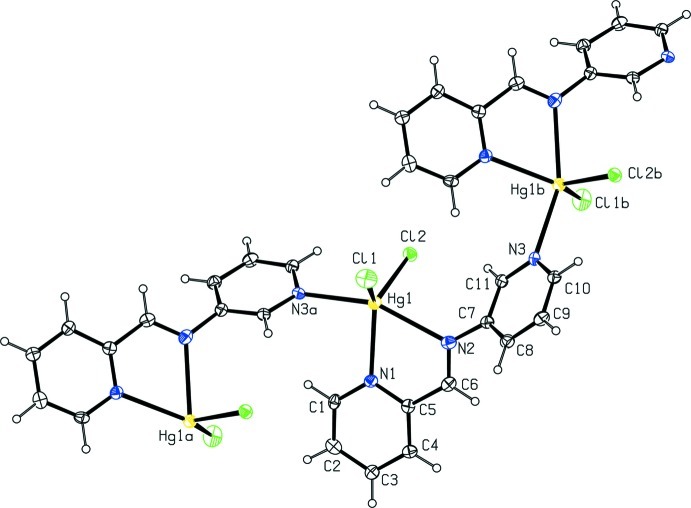
A view of the structure of the title complex, with displacement ellipsoids drawn at the 50% probability level [symmetry codes: (a) 1/2 - x, -1/2 + y, 1/2 - z; (b) 1/2 - x, -1/2 + y, 1/2 - z].

## supplementary crystallographic information

### Comment

Many studies has recently been focused on coordination polymers due to their useful properties applicable to catalysis, chirality, conductivity, luminescence (Moulton & Zaworotko, 2001). Nitrogen heterocyclic ligands have been employed in the design and synthesis of many novel coordination polymers (Fei *et al.*, 2000). Herewith we report the synthesis and crystal structure of a novel Hg(II) complex based on pyridin-3-ylpyridin-2-ylmethyleneamine (PyPy).

The asymmetric unit of the title polymeric complex, consisting of one Hg(II) ion, one PyPy ligand and two chloride anions, is shown in Fig. 1. The coordination geometry around Hg(II) is a distorted trigonal–bipyramidal geometry, with the Hg (II) ion being surrounded by two Cl, two N atoms from one PyPy ligand and one N atom from adjacent PyPy ligand. The structural index τ, (Dehghanpour *et al.*, 2012) which is a measure of trigonal distortion, is 0.59 for the title structure indicating a distorted trigonal–bipyramidal environment of Hg(II).

The interplanar angles between the chelate ring (N1—C5—C6—N2) and pyridine ring (N1—C1—C2—C3—C4—C5) is 0.92 (3)° and interplanar angles between the two pyridine rings in the ligand (N1—C1—C2—C3—C4—C5 ring and N3—C11—C7—C8—C9—C10 ring) is 50.0 (2)°. Each PyPy ligand has been chelate HgCl_2_ unit (*via* N, N' atoms) and also bridge to another HgCl_2_ unit (with N" atom), resulting into a chain propagated in [010].

These chains interact *via*π–π interactions between adjacent pyridine ringe (N3/C7—C11) related by inversion center, and the distance between their centroids is equal to 3.529 (9) Å. The C—H···Cl interactions (Table 1) are also observed in the crystal structure.

### Experimental

The title complex was prepared by the reaction of HgCl_2_ (27.1 mg, 0.1 mmol) and pyridin-3-ylpyridin-2-ylmethyleneamine (18.3 mg, 0.1 mmol) in 25 ml of acetonitrile at room temperature. The solution was allowed to stand at room temperature and yellow crystals of the title compound suitable for X-ray analysis precipitated within few days.

### Refinement

H atoms were placed in calculated positions with C—H = 0.95 Å, and included in the refinement in a riding-motion approximation, with *U*_iso_(H)= 1.2*U*_eq_(C).

### Crystal data

**Table:**

[HgCl_2_(C_11_H_9_N_3_)]	*F*(000) = 840
*M**_r_* = 454.70	*D*_x_ = 2.412 Mg m^−^^3^
Monoclinic, *P*2_1_/*n*	Mo *K*α radiation, λ = 0.71073 Å
Hall symbol: -P 2yn	Cell parameters from 8560 reflections
*a* = 7.5645 (5) Å	θ = 3.0–27.5°
*b* = 13.1057 (9) Å	µ = 12.70 mm^−^^1^
*c* = 12.7017 (5) Å	*T* = 150 K
β = 96.077 (4)°	Plate, yellow
*V* = 1252.15 (13) Å^3^	0.15 × 0.08 × 0.02 mm
*Z* = 4	

### Data collection

**Table:**

Nonius KappaCCD diffractometer	2837 independent reflections
Radiation source: fine-focus sealed tube	2164 reflections with *I* > 2σ(*I*)
Graphite monochromator	*R*_int_ = 0.059
Detector resolution: 9 pixels mm^-1^	θ_max_ = 27.5°, θ_min_ = 3.0°
φ scans and ω scans with κ offsets	*h* = −9→9
Absorption correction: multi-scan (*SORTAV*; Blessing, 1995)	*k* = −15→16
*T*_min_ = 0.566, *T*_max_ = 0.889	*l* = −16→16
8560 measured reflections	

### Refinement

**Table:**

Refinement on *F*^2^	Primary atom site location: structure-invariant direct methods
Least-squares matrix: full	Secondary atom site location: difference Fourier map
*R*[*F*^2^ > 2σ(*F*^2^)] = 0.042	Hydrogen site location: inferred from neighbouring sites
*wR*(*F*^2^) = 0.116	H-atom parameters constrained
*S* = 1.09	*w* = 1/[σ^2^(*F*_o_^2^) + (0.0613*P*)^2^] where *P* = (*F*_o_^2^ + 2*F*_c_^2^)/3
2837 reflections	(Δ/σ)_max_ = 0.002
154 parameters	Δρ_max_ = 2.45 e Å^−^^3^
0 restraints	Δρ_min_ = −3.10 e Å^−^^3^

### Special details

**Table:**

Geometry. All e.s.d.'s (except the e.s.d. in the dihedral angle between two l.s. planes) are estimated using the full covariance matrix. The cell e.s.d.'s are taken into account individually in the estimation of e.s.d.'s in distances, angles and torsion angles; correlations between e.s.d.'s in cell parameters are only used when they are defined by crystal symmetry. An approximate (isotropic) treatment of cell e.s.d.'s is used for estimating e.s.d.'s involving l.s. planes.
Refinement. Refinement of *F*^2^ against ALL reflections. The weighted *R*-factor *wR* and goodness of fit *S* are based on *F*^2^, conventional *R*-factors *R* are based on *F*, with *F* set to zero for negative *F*^2^. The threshold expression of *F*^2^ > σ(*F*^2^) is used only for calculating *R*-factors(gt) *etc*. and is not relevant to the choice of reflections for refinement. *R*-factors based on *F*^2^ are statistically about twice as large as those based on *F*, and *R*- factors based on ALL data will be even larger.

### Fractional atomic coordinates and isotropic or equivalent isotropic displacement parameters (Å^2^)

**Table:**

	*x*	*y*	*z*	*U*_iso_*/*U*_eq_	
Hg1	0.26173 (3)	0.14916 (2)	0.252995 (19)	0.02692 (14)	
Cl1	−0.0045 (3)	0.16859 (19)	0.13060 (18)	0.0509 (6)	
Cl2	0.5101 (2)	0.27229 (14)	0.25673 (14)	0.0296 (4)	
N1	0.2620 (7)	0.0445 (4)	0.4086 (4)	0.0249 (13)	
N2	0.1391 (8)	0.2435 (5)	0.4036 (4)	0.0295 (14)	
N3	0.0833 (8)	0.5128 (4)	0.3314 (4)	0.0261 (13)	
C1	0.3228 (9)	−0.0520 (6)	0.4171 (5)	0.0275 (16)	
H1A	0.3673	−0.0821	0.3572	0.033*	
C2	0.3237 (10)	−0.1096 (6)	0.5084 (6)	0.0316 (18)	
H2A	0.3702	−0.1770	0.5113	0.038*	
C3	0.2552 (9)	−0.0670 (6)	0.5963 (5)	0.0293 (16)	
H3A	0.2501	−0.1055	0.6592	0.035*	
C4	0.1954 (9)	0.0319 (6)	0.5895 (6)	0.0272 (16)	
H4A	0.1513	0.0636	0.6487	0.033*	
C5	0.1999 (9)	0.0854 (6)	0.4950 (5)	0.0257 (16)	
C6	0.1330 (9)	0.1915 (6)	0.4877 (5)	0.0284 (16)	
H6A	0.0849	0.2213	0.5467	0.034*	
C7	0.0619 (11)	0.3439 (5)	0.3945 (6)	0.0299 (17)	
C8	−0.1065 (10)	0.3615 (6)	0.4214 (6)	0.0308 (17)	
H8A	−0.1701	0.3091	0.4527	0.037*	
C9	−0.1826 (10)	0.4570 (6)	0.4022 (6)	0.0319 (18)	
H9A	−0.2995	0.4714	0.4191	0.038*	
C10	−0.0815 (9)	0.5309 (6)	0.3574 (5)	0.0254 (16)	
H10A	−0.1311	0.5969	0.3447	0.030*	
C11	0.1534 (10)	0.4210 (6)	0.3503 (5)	0.0284 (16)	
H11A	0.2703	0.4080	0.3328	0.034*	

### Atomic displacement parameters (Å^2^)

**Table:**

	*U*^11^	*U*^22^	*U*^33^	*U*^12^	*U*^13^	*U*^23^
Hg1	0.02627 (19)	0.0269 (2)	0.0285 (2)	−0.00027 (12)	0.00688 (13)	−0.00040 (11)
Cl1	0.0333 (12)	0.0627 (16)	0.0536 (14)	−0.0076 (10)	−0.0097 (9)	0.0062 (11)
Cl2	0.0272 (9)	0.0266 (10)	0.0357 (9)	−0.0027 (7)	0.0071 (7)	−0.0009 (8)
N1	0.020 (3)	0.028 (4)	0.027 (3)	−0.006 (3)	0.006 (2)	−0.001 (3)
N2	0.027 (3)	0.035 (4)	0.026 (3)	−0.008 (3)	0.001 (2)	0.001 (3)
N3	0.030 (3)	0.022 (3)	0.027 (3)	0.003 (3)	0.007 (2)	−0.003 (3)
C1	0.022 (4)	0.030 (4)	0.031 (4)	0.000 (3)	0.006 (3)	−0.008 (3)
C2	0.020 (4)	0.032 (5)	0.041 (4)	−0.001 (3)	−0.005 (3)	0.004 (3)
C3	0.027 (4)	0.033 (4)	0.028 (4)	0.000 (3)	0.001 (3)	−0.001 (3)
C4	0.023 (4)	0.033 (5)	0.026 (4)	−0.003 (3)	0.002 (3)	0.002 (3)
C5	0.019 (4)	0.029 (4)	0.029 (4)	0.001 (3)	0.002 (3)	−0.001 (3)
C6	0.019 (4)	0.038 (5)	0.029 (4)	−0.001 (3)	0.003 (3)	−0.002 (4)
C7	0.038 (4)	0.027 (4)	0.025 (4)	0.004 (3)	0.007 (3)	0.003 (3)
C8	0.031 (4)	0.033 (5)	0.028 (4)	−0.006 (3)	0.005 (3)	−0.002 (3)
C9	0.023 (4)	0.042 (5)	0.031 (4)	−0.001 (3)	0.006 (3)	−0.001 (4)
C10	0.028 (4)	0.029 (4)	0.018 (3)	0.004 (3)	−0.004 (3)	0.002 (3)
C11	0.031 (4)	0.025 (4)	0.030 (4)	0.004 (3)	0.011 (3)	0.000 (3)

### Geometric parameters (Å, º)

**Table:**

Hg1—N1	2.406 (5)	C2—H2A	0.9500
Hg1—Cl1	2.424 (2)	C3—C4	1.373 (11)
Hg1—N3^i^	2.445 (6)	C3—H3A	0.9500
Hg1—Cl2	2.4732 (17)	C4—C5	1.393 (10)
Hg1—N2	2.535 (6)	C4—H4A	0.9500
N1—C1	1.347 (9)	C5—C6	1.480 (11)
N1—C5	1.349 (8)	C6—H6A	0.9500
N2—C6	1.272 (9)	C7—C8	1.373 (11)
N2—C7	1.438 (9)	C7—C11	1.378 (10)
N3—C11	1.327 (9)	C8—C9	1.388 (11)
N3—C10	1.345 (8)	C8—H8A	0.9500
N3—Hg1^ii^	2.445 (6)	C9—C10	1.393 (10)
C1—C2	1.384 (10)	C9—H9A	0.9500
C1—H1A	0.9500	C10—H10A	0.9500
C2—C3	1.395 (10)	C11—H11A	0.9500
			
N1—Hg1—Cl1	121.03 (14)	C4—C3—H3A	120.8
N1—Hg1—N3^i^	89.13 (19)	C2—C3—H3A	120.8
Cl1—Hg1—N3^i^	101.53 (15)	C3—C4—C5	119.4 (7)
N1—Hg1—Cl2	114.93 (14)	C3—C4—H4A	120.3
Cl1—Hg1—Cl2	121.44 (7)	C5—C4—H4A	120.3
N3^i^—Hg1—Cl2	94.98 (14)	N1—C5—C4	122.9 (7)
N1—Hg1—N2	68.1 (2)	N1—C5—C6	118.0 (6)
Cl1—Hg1—N2	95.00 (15)	C4—C5—C6	119.1 (6)
N3^i^—Hg1—N2	156.61 (19)	N2—C6—C5	120.9 (6)
Cl2—Hg1—N2	90.30 (14)	N2—C6—H6A	119.6
C1—N1—C5	117.0 (6)	C5—C6—H6A	119.6
C1—N1—Hg1	124.9 (4)	C8—C7—C11	119.8 (7)
C5—N1—Hg1	118.2 (5)	C8—C7—N2	120.9 (7)
C6—N2—C7	120.5 (6)	C11—C7—N2	119.1 (6)
C6—N2—Hg1	114.9 (5)	C7—C8—C9	119.2 (7)
C7—N2—Hg1	124.3 (4)	C7—C8—H8A	120.4
C11—N3—C10	118.6 (6)	C9—C8—H8A	120.4
C11—N3—Hg1^ii^	122.8 (5)	C8—C9—C10	117.6 (7)
C10—N3—Hg1^ii^	118.6 (5)	C8—C9—H9A	121.2
N1—C1—C2	123.4 (6)	C10—C9—H9A	121.2
N1—C1—H1A	118.3	N3—C10—C9	122.8 (7)
C2—C1—H1A	118.3	N3—C10—H10A	118.6
C1—C2—C3	118.9 (7)	C9—C10—H10A	118.6
C1—C2—H2A	120.6	N3—C11—C7	122.1 (7)
C3—C2—H2A	120.6	N3—C11—H11A	119.0
C4—C3—C2	118.4 (7)	C7—C11—H11A	119.0

Symmetry codes: (i) −*x*+1/2, *y*−1/2, −*z*+1/2; (ii) −*x*+1/2, *y*+1/2, −*z*+1/2.

### Hydrogen-bond geometry (Å, º)

**Table:**

*D*—H···*A*	*D*—H	H···*A*	*D*···*A*	*D*—H···*A*
C4—H4*A*···Cl2^iii^	0.95	2.82	3.700 (8)	154
C6—H6*A*···Cl2^iii^	0.95	2.79	3.666 (7)	154
C10—H10*A*···Cl2^ii^	0.95	2.83	3.545 (8)	132

Symmetry codes: (ii) −*x*+1/2, *y*+1/2, −*z*+1/2; (iii) *x*−1/2, −*y*+1/2, *z*+1/2.
